# Empyema necessitans due to *Nocardia farcinica*

**DOI:** 10.1016/j.idcr.2022.e01545

**Published:** 2022-06-22

**Authors:** Kazuhiro Ishikawa, Nobuyoshi Mori

**Affiliations:** Department of Infectious Diseases, St. Luke’s International Hospital, Tokyo, Japan

**Keywords:** *Nocardia farcinica*, Empyema necessitans, Liver cirrhosis

## Abstract

An 84-year-old female with severe liver cirrhosis due to hepatitis C presented with a progressive bulging of the anterior chest wall for two weeks. On examination, 7 cm × 7 cm × 5 cm large subcutaneous mass was on the anterior chest wall and contrast enhanced computed tomography of the chest demonstrated loculated empyema with extension through the chest wall, into the anterior with rib destruction. Microscopic appearance of the abscess revealed filamentous branching rods, and eventually Nocardia farcinica was identified using Matrix-Assisted Laser Desorption Ionization–Time of Flight. The patient was successfully treated with trimethoprim/sulfamethoxazole and subcutaneous debridement. In general, Mycobacterium tuberculosis and Actinomyces spp. are the most common causative pathogens of empyema necessitans, and cases of Nocardia spp. are rarely seen. Clinicians should pay attention to the possibility of nocardial empyema necessitans in immunocompromised patients.

An 84-year-old female with Child-Pugh class C liver cirrhosis due to hepatitis C presented with a progressive bulging of the anterior chest wall for two weeks. She has not been exposed to soil, gardening, and construction sites. She had not gotten any medical procedures. She denied fevers or chills, productive cough, hemoptysis, changes in weight, or loss of appetite. On examination, there was no dental cavity or ulcer in the oral mucosa. Heart auscultation revealed a regular rate and rhythm without a murmur appreciated. Large subcutaneous mass was on the anterior chest wall (Fig. 1, arrow). No axillary lymphadenopathy was observed. Laboratory data revealed a white blood cell 4600/μL (neutrophil 84%), hemoglobin of 9.9 g/dL, platelet count of 158 × 10^3^/μL. Other bloodwork revealed aspartate aminotransferase of 27 U/L, alanine aminotransferase of 13 U/L, total bilirubin of 0.7 mg/dL, creatinine of 0.5 mg/dL and C-reactive protein of 7.1 mg/dl. Contrast-enhanced computed tomography of the chest demonstrated 7 cm x 7 cm x 5 cm loculated empyema with extension through the chest wall, into the anterior chest wall with rib destruction (Fig. 2). Microscopic appearance of the abscess revealed filamentous branching rods (Fig. 3, A: Gram stain, B: Kinyoun stain). *Nocardia farcinica* was identified using Matrix-Assisted Laser Desorption Ionization–Time of Flight. The susceptibility of antibiotics to *Nocardia farcinica* were shown in Table 1. She was successfully treated with trimethoprim/sulfamethoxazole 5 mg per kg per dose 3 times a day and subcutaneous debridement and eventually the empyema necessitans disappeared. But on the 81st day of hospitalization, the patient died of acute respiratory distress syndrome due to aspiration pneumonia. In general, *Mycobacterium tuberculosis* and *Actinomyces* spp*.* are the most common causative pathogens of empyema necessitans [1], and cases of *Nocardia* spp. are rarely seen [2]. In general, the risk factor of *Nocardia spp.* is a preceding history of contaminated water or soil ingestion, and the patient did not respond to beta-lactam. *N. farcinica* is a clinically aggressive infection, particularly in immunocompromised patients such as lung abscess [3] and brain abscess [4]. Clinicians should pay attention to the possibility of nocardial empyema necessitans in immunocompromised patients.

**Fig. 1:**
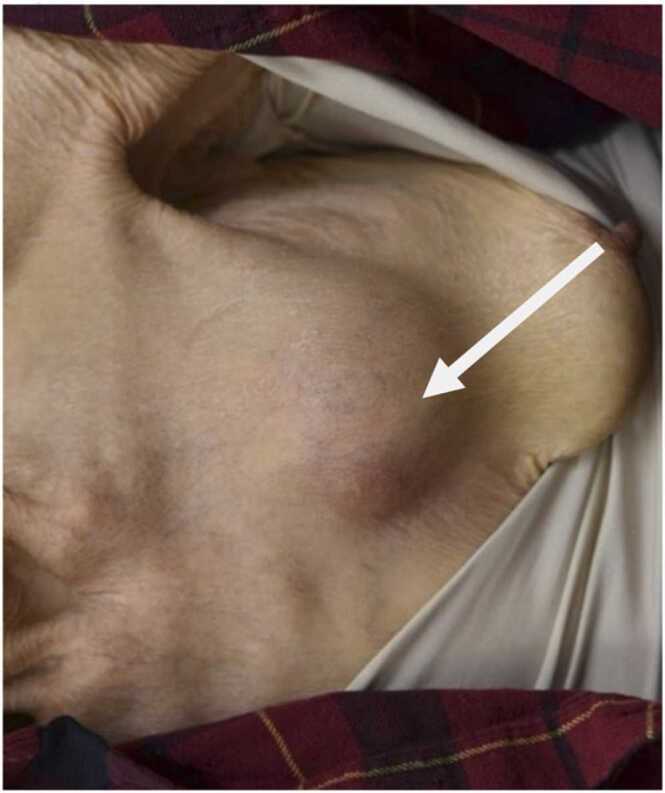
On the anterior chest, the subcutaneous abscess (arrow) was found on the right side of the left breast.

**Fig. 2:**
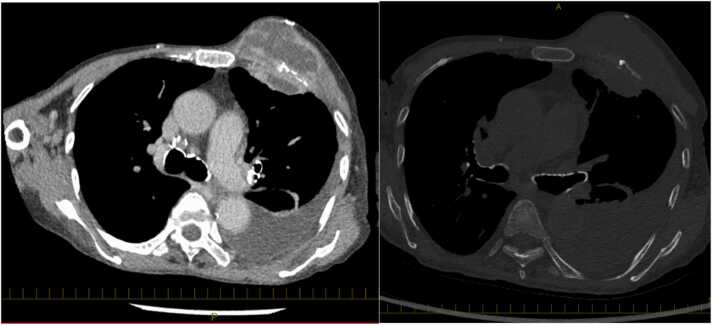
Contrast-enhanced computed tomography (CT) showed loculated empyema with extension through the chest wall into the anterior chest (left). Bone window showed left rib destruction (right).

**Fig. 3:**
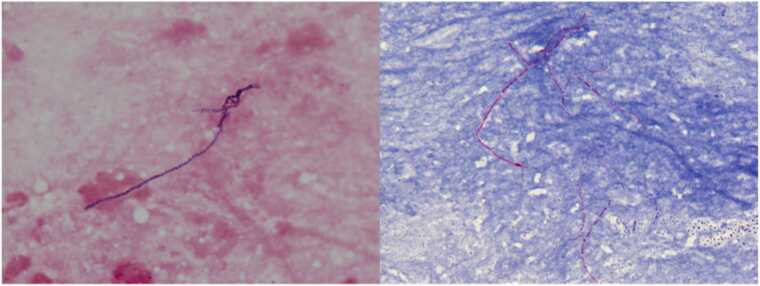
Gram stain (left) revealed filamentous gram-positive rods. We evaluated Kinyoun stain (right) revealed thin, slender, acid-fast, beaded and branched bacilli after identification of the Nocardia farcinica.

**Table 1:** Results of susceptibility testing; using the broth microdilution method of *Nocardia farcinica.*

Antimicrobials	MIC (μg/mL)	Susceptibility	*Breakpoint (μg/mL)
Amikacin	2	S	S: ≤ 8 R: ≥ 16
Ceftriaxone	> 64	R	S: ≤ 8 I: 16–32 R: ≥ 64
Imipenem	16	R	S: ≤ 4 I: 8 R: ≥ 16
Trimethoprim/sulfamethoxazole	9.5/0.5	S	S: ≤ 38/2 R: ≥ 76/4
Cefotaxime	> 64	R	S: ≤ 8 I: 16–32 R: ≥ 64
Minocycline	2	I	S: ≤ 1 I: 2–4 R: ≥ 8
Linezolid	4	S	< =8

* Reference from Clinical and Laboratory Standards Institute (CLSI) M24-A2.

## Author’s contributions

The manuscript was seen and approved by all the authors and is not under consideration elsewhere. All the authors contributed to the work in this report. KI collected clinical data and wrote the initial draft of the manuscript. NM supervised and edited the manuscript. The author(s) read and approved the final manuscript.

## Funding

I swear that I have not received any funding.

## Consent

The patient described in this paper has given written, informed consent to publishher case, radiographic images, and pathological reports.

## Ethical approval

The research in this paper was conducted ethically in accordance with the World Medical Association Declaration of Helsinki.

## Conflict of interest

I swear that I have no conflicts of interest.
